# Impact of healthcare inequities on survival in Mexican patients with metastatic renal cell carcinoma

**DOI:** 10.3389/fonc.2023.1229016

**Published:** 2023-11-17

**Authors:** Maria T. Bourlon, Yuly A. Remolina-Bonilla, Aldo A. Acosta-Medina, Bruno I. Saldivar-Oviedo, Antonio Perez-Silva, Nayeli Martinez-Ibarra, Francisco Javier Castro-Alonso, Ana E. Martín-Aguilar, Samuel Rivera-Rivera, Fernando Mota-Rivero, Perla Pérez-Pérez, María G. Díaz-Alvarado, José M. Ruiz-Morales, Saúl Campos-Gómez, Bertha Alejandra Martinez-Cannon, Elaine T. Lam, Nora Sobrevilla-Moreno

**Affiliations:** ^1^ Department of Hematology and Oncology, Instituto Nacional de Ciencias Médicas y Nutrición Salvador Zubirán, Mexico City, Mexico; ^2^ Department of Medical Oncology, Centro Médico Nacional Siglo XXI, Mexico City, Mexico; ^3^ Department of Medical Oncology, Centro Médico Nacional 20 Noviembre, Mexico City, Mexico; ^4^ Comprehensive Cancer Center, Médica Sur, Mexico City, Mexico; ^5^ Statal Oncologic Center, Instituto de Seguridad Social del Estado de México y Municipios, Toluca, Mexico; ^6^ Department of Hematology and Medical Oncology, University of Colorado Cancer Center, Aurora, CO, United States; ^7^ Department of Medical Oncology, Instituto Nacional de Cancerología, Mexico City, Mexico

**Keywords:** metastatic renal cell carcinoma, Mexico, Latin America, resource-limited setting, survival, kidney cancer

## Abstract

**Introduction:**

The survival of patients with metastatic renal cell carcinoma (mRCC) has improved dramatically due to novel systemic treatments. However, mRCC mortality continues to rise in Latin America.

**Methods:**

A retrospective, multicenter study of patients diagnosed with mRCC between 2010-2018 in Mexico City was conducted. The aim of the study was to evaluate the impact of healthcare insurance on access to treatment and survival in patients with mRCC.

**Results:**

Among 924 patients, 55.4%, 42.6%, and 1.9% had no insurance (NI), social security, (SS) and private insurance (PI), respectively. *De novo* metastatic disease was more common in NI patients (70.9%) compared to SS (47.2%) and PI (55.6%) patients (p<0.001). According to IMDC Prognostic Index, 20.2% were classified as favorable, 49% as intermediate, and 30.8% as poor-risk disease. Access to systemic treatment differed by healthcare insurance: 36.1%, 99.5%, and 100% for the NI, SS, and PI patients, respectively (p<0.001). NI patients received fewer lines of treatment, with 24.8% receiving only one line of treatment (p<0.001). Median overall survival (OS) was 13.9 months for NI, 98.9 months for SS, and 147.6 months for NI patients (p<0.001). In multivariate analysis, NI status, brain metastases, sarcomatoid features, bone metastases, no treatment were significantly associated with worse OS.

**Conclusion:**

OS in mRCC was affected by insurance availability in this resource-limited cohort of Mexican patients. These results underscore the need for effective strategies to achieve equitable healthcare access in an era of effective, yet costly systemic treatments.

## Introduction

Globally, renal cell carcinoma (RCC) is the 15th most common cancer (1), and its incidence has continued to increase over the last decades, with the greatest rise in Latin America (2, 3). This trend is partly explained by the increased use of imaging studies and the subsequent identification of incidental renal masses, as well as, by an increase in the prevalence of known risk factors for this neoplasm, such as obesity, hypertension, and western-type diet (4).

Most cases of RCC are currently diagnosed in high-income countries where an increase in stage I disease and a decrease in stages II-IV have been reported. Nonetheless, approximately 15% and 22% of patients are diagnosed with locally advanced (laRCC) or metastatic (mRCC) RCC, respectively (5, 6). Despite a continued increase in RCC incidence, 5-year overall survival (OS) has doubled from 34% in the 1950’s to 73% in 2000’s (7). This is in large part attributable to increased rates of early-stage disease at diagnosis and curative treatment in this setting (8). Likewise, a continued improvement in survival outcomes of patients with laRCC and mRCC has also been achieved due to novel systemic treatments including tyrosine-kinase inhibitors (TKIs) (9, 10) immune-checkpoint inhibitors (11, 12), and their combination (13, 14).

Unfortunately, RCC mortality has continued rising in low- and middle-income countries (LMICs), including most of Latin America (15). This phenomenon can be partly explained by a recent increase in reporting in population-based cancer registries and by limited access to effective treatments (16). Mexico is classified as an upper middle-income country (17) with a high human development index (18), however, significant inequities in resource distribution have led to a high prevalence of poverty and lack of universal access to healthcare services. The Mexican healthcare system comprises three sectors: i) Social Security (SS) which provides full coverage of healthcare services to tax-payers and their families according to the Prescription Drug Formularies (not necessarily covering current standard of care treatments, usually considered high-cost therapies), ii) Private insurance (PI), which covers those with purchasing power and represents less than 10% of the population, and iii) No insurance (NI) in which the Mexican Department of Health sets stipends for specific cancers in the population according to monthly income (systemic treatment for mRCC is not included) (19). Consequently, for Mexicans with NI, a high proportion of healthcare costs are paid out-of-pocket, limiting access to treatment. This segmented, complex Mexican healthcare system gives place to intrinsic disparities in access and quality of healthcare. Thus, the aim of this study was to evaluate the effect of healthcare insurance on systemic treatment received and survival outcomes in patients with mRCC in Mexico City.

## Materials and methods

### Study design and data collection

A retrospective, multicenter study of patients diagnosed with mRCC in the Mexico City Metropolitan Area between 2010 to 2018 was conducted. Eligible patients were over 18 years of age, had histologic confirmation of RCC, and evidence of metastatic disease by imaging studies. Patients without available medical records were excluded. Demographic, clinical, treatment, prognostic, and outcomes data were collected from electronic and/or physical medical records. Demographic and clinical characteristics included age, gender, distance from the place of residence to the healthcare center, type of healthcare insurance, histology, International mRCC Database Consortium (IMDC) Prognostic Index, and number and site of metastases. Data related to treatment included surgical interventions (nephrectomy or metastasectomy), radiotherapy, systemic therapy (type and number of systemic agents received), and time from the metastatic disease diagnosis to time treatment initiation.

### Ethical considerations

The study was conducted in accordance with the Declaration of Helsinki and was approved by Institutional and Ethics Review Boards within all participating institutions: Instituto Nacional de Ciencias Médicas y Nutrición Salvador Zubirán, Centro Médico Nacional Siglo XXI, Centro Médico Nacional 20 Noviembre, Médica Sur, Instituto de Seguridad Social del Estado de México y Municipios, and Instituto Nacional de Cancerología. All participating institutions are part of a national research initiative, the Collaborative Oncology Research Group of Mexico (GCIMO), Genitourinary Oncology section, created and supported by the Mexican Society of Oncology (SMeO).

### Statistical analysis

Descriptive statistics were used to analyze demographic, clinical, treatment, and prognostic characteristics. The patients included in this study were categorized according to their type of healthcare insurance in three groups: SS, PI, and NI. Associations between variables and healthcare insurance were evaluated with the Chi-square test for Independence or Fisher’s exact test, accordingly. OS was measured from time of diagnosis of mRCC to the date of last follow-up or death. Patients lost to follow-up were censored from the OS analysis. Kaplan-Meier curves and log-rank test were used to estimate and compare OS according to healthcare insurance status. A Cox regression model for multivariate survival analysis was performed. P values <0.05 were considered statistically significant. The software used for data analysis was SPSS version 25 (IBM Corp, Armonk, NY).

## Results

### Patients’ characteristics

A total of 924 patients met inclusion criteria. Median age at diagnosis was 58 years (range 18-100) and 67.7% of patients were men (n=626). Of these, 55.4% (n=512), 42.6% (n=394) and 1.9% (n=18) were in NI, SS and PI health subsystems, respectively. Characteristics of the overall population and according to the healthcare insurance are shown in **Table 1**. All participating centers were located within the Mexico City Metropolitan Area; nonetheless, 25.1% (n=232) of patients lived outside of this region, with at least 20.3% of patients experiencing travel intervals exceeding 4 hours to receive specialized medical care. When the patients were classified according to healthcare insurance, those with SS (88.6%) and PI (100%) were more likely to live in Mexico City compared with NI (62.7%) population (p<0.001).

**Table 1:** Characteristics of overall population and according to healthcare insurance.

Characteristic	Overall population n = 924 (%)	No insurance n = 512 (%)	Social Security n = 394 (%)	Private insurance n = 18 (%)	p-value
Median age (years)	58	57	58	58	0.129
Gender - Female - Male	298 (32.3) 626 (67.7)	158 (30.9) 354 (69.1)	135 (34.3) 259 (65.7)	5 (27.8) 13 (72.2)	0.509
Mexico City Metropolitan Area	688 (74.5)	321 (62.7)	349 (88.6)	18 (100)	**<0.001**
*De novo* metastatic disease	559 (60.5)	363 (70.9)	186 (47.2)	10 (55.6)	**<0.001**
Histology - Clear cell - Papillary - Chromophobe	728 (78.8) 22 (2.4) 15 (1.6)	347 (90.6) 11 (2.9) 7 (1.8)	366 (93.8) 10 (2.6) 7 (1.8)	15 (83.3) 1 (5.6) 1 (5.6)	0.277
Sarcomatoid features	73 (7.9)	49 (13.1)	21 (5.4)	3 (17.6)	**0.001**
Numbers of sites of metastases - 1 - 2-3 - > 3	377 (40.8) 459 (49.7) 88 (9.5)	218 (42.6) 245 (47.9) 49 (9.5)	155 (39.3) 205 (52) 44 (8.5)	4 (22.2) 9 (50) 5 (27.8)	**0.049**
Sites of metastases - Lung - Bone - Lymph nodes - Liver - Brain - Soft Tissue	708 (76.6) 269 (29.1) 267 (28.9) 193 (20.9) 114 (12.3) 98 (10.6)	397 (77.5) 144 (28.1) 162 (31.6) 101 (19.7) 54 (10.5) 55 (10.7)	294 (74.6) 118 (29.9) 98 (24.9) 88 (22.3) 51 (12.9) 43 (10.9)	17 (94.4) 7 (38.9) 7 (38.9) 4 (22.2) 9 (50) 0	0.116 0.546 0.054 0.626 **<0.001** 0.335
IMDC Prognostic Index - Favorable - Intermediate - Poor	157 (20.2) 381 (49) 239 (30.8)	23 (6.1) 165 (43.9) 188 (50)	131 (33.7) 210 (54) 48 (12.3)	3 (25) 6 (50) 3 (25)	**<0.001**
Nephrectomy	633 (68.5)	300 (58.6)	316 (80.2)	17 (94.4)	**<0.001**
Metastasectomy	106 (11.5)	53 (10.4)	47 (11.9)	6 (33.3)	**0.01**
Palliative Radiotherapy	269 (29.1)	147 (28.7)	117 (29.7)	5 (27.8)	0.942
Bisphosphonates	60 (6.5)	18 (3.5)	39 (9.9)	3 (16.7)	**<0.001**
Systemic treatment - Yes	595 (64.4)	185 (36.1)	392 (99.5)	18 (100)	**<0.001**
Lines of systemic treatment - 0 - 1 - 2 - 3 - 4	329 (35.6) 384 (41.6) 143 (15.5) 53 (5.7) 15 (1.6)	327 (63.9) 127 (24.8) 40 (7.8) 14 (2.7) 4 (0.8)	2 (0.5) 247 (62.7) 100 (25.4) 36 (9.1) 9 (2.3)	0 (0) 10 (55.6) 3 (16.7) 3 (16.7) 2 (11)	**<0.001**

Bold values are those that reached statistical significance.

In terms of histology, clear cell RCC represented 78.8% (n=728) of cases; while 2.4% (n=22), 1.6% (n=15), and 17.2% (n=159) were papillary, chromophobe, and other/unknown histology, respectively. Sarcomatoid features were observed in 7.9% (n=73) of patients; this characteristic was less frequent in the SS population (p = 0.001). Within the overall cohort, 39.5% (n=365) were initially diagnosed with stage I-III RCC, with a median time to progression to metastatic disease of 20 months (95% confidence interval (CI), 16.2 - 24), with no statistically significant difference in time to metastatic disease between groups according to healthcare insurance. *De novo* metastatic disease was observed in 60.5% (n=559) of the overall population but was more common in NI patients (70.9%) compared with SS (47.2%) and PI (55.6%) patients (p<0.001).

Data for evaluating IMDC Prognostic Index was available in 84.1% (n=777) of the cohort, 20.2% (n=157) were classified as favorable-risk, 49% (n=381) as intermediate-risk, and 30.8% (n=239) as poor-risk disease. According to insurance status, statistically significant differences were found in IMDC risk groups, with 50% of the NI patients having poor risk and only 6% favorable risk disease. In the SS (54%) and PI (50%) subgroups the majority of patients were classified as intermediate risk (p<0.001).

The most common sites of metastases included lung (76.6%), bone (29.1%), and lymph nodes (28.9%). At the time of diagnosis, 40.8% of patients had metastatic disease confined to a single site, while 59.2% had ≥2 sites involved. The PI population had higher metastatic burden, 77.8% of patients had ≥2 metastatic sites and brain metastases (50%) compared to the SS (60.5% and 12.9%, respectively) and NI (57.4% and 10.5%, respectively) subgroups (p = 0.049 and p<0.001, respectively).

### Access to therapy and treatment

Nephrectomy was performed in 68.5% (n=633) of the overall population; PI (94.4%) and SS (80.2%) patients underwent nephrectomy more frequently than NI patients (58.6%) (p<0.001). Metastasectomy was performed in 11.5% of patients (n=106), especially in the PI group, with 33.3% of patients received this intervention (p = 0.01). Almost one third (n = 269) of the overall cohort received palliative radiotherapy with no differences between groups. Treatment with antiresorptive bone agents such as bisphosphonates occurred only in 6.5% (n=60) of the overall population.

Systemic therapy for mRCC was initiated in 64.4% (n=595) of the overall cohort; however, access to systemic treatment markedly differed among healthcare insurance: 36.1% (n=185), 99.5% (n=392), and 100% (n=18) for NI, SS, and PI groups, respectively (p < 0.001). Median time from diagnosis of metastatic disease to therapy initiation was 2 months (range 0-129 months) for the overall cohort and was not statistically different between healthcare insurance subgroups (p = 0.499). In the overall cohort, 41.6% (n=384) received only one line of therapy, 16.2% (n=150) two lines, 5.1% (n=47) three lines, and 1.5% (n=14) four lines. Among SS patients, 61.7% received first-line treatment and 26.7% second-line treatment. NI patients received fewer lines of treatment (p<0.001), with 24.8% of them receiving only a one line of systemic treatment and 63.9% receiving no systemic treatment at all. Patients who received third- and fourth-line therapy were most frequently PI patients.

Sunitinib was the most common choice of first-line therapy: 57.1% of patients received sunitinib, 17.3% pazopanib, 9.9% sorafenib, and 9.6% interferon. The treatment regimens more frequently used in second-line setting included sorafenib (33.2%), sunitinib (15.2%), and pazopanib (14.7%). In the third-line setting, 18% patients received sorafenib, 18% everolimus, and 16.4% nivolumab. Sunitinib was the agent that SS and NI patients received most frequently in first-line setting (65.1% and 44.3%, respectively), while PI patients were more frequently treated with pazopanib (55.6%). In the second-line setting, sorafenib was most frequently received in SS patients (40.3%), while pazopanib continued to be the most prescribed treatment for PI patients (50%). Among NI patients, only 18.5% received sorafenib and up to 40% received other therapies not included among the most effective for this disease. The therapies received by patients according to the type of healthcare insurance were statistically different and are shown in **Table 2**.

**Table 2:** Systemic treatment received according to insurance status and treatment line.

1st line	No insurance	Social Security	Private insurance	p-value
Sunitinib	44.3%	65.1%	16.7%	<0.001*
Sorafenib	0.5%	14.5%	5.6%	
Pazopanib	25.9%	11.5%	55.6%	
Interferon (INF)	22.2%	4.1%	0	
Bevacizumab/INF	0	3.8%	5.6%	
Clinical Trial	1.6%	0.5%	0	
Other	5.5%	0.5%	16.5%	
2nd line				
Sorafenib	18.5%	40.3%	0	<0.001*
Sunitinib	11.1%	16.8%	12.5%	
Pazopanib	9.3%	14.8%	50%	
Everolimus	11.1%	14.1%	12.5	
Bevacizumab/INF	0	8.1%	0	
Nivolumab	9.3%	0	25%	
Other	40.7%	5.9%	0	
3rd line				
Nivolumab	33.3%	9.1%	40%	0.025^
Everolimus	0	22.7%	20%	
Bevacizumab/INF	0	6.8%	20%	
Clinical Trial	0	0	20%	
Sorafenib	8.3%	22.7%	0	
Pazopanib	33.3%	13.6%	0	
Other	25.1%	25.1%	0	
4th line				
Sunitinib	0	23.7%	50%	0.178^
Nivolumab	0	0	50%	
Everolimus	0	18.2%	0	
Sorafenib	0	18.2%	0	
Axitinib	100%	0	0	
Other	0	39.9%	0	

*Chi-square test.

^Fisher’s exact test.

Bold values are those that reached statistical significance.

**Figure 1:**
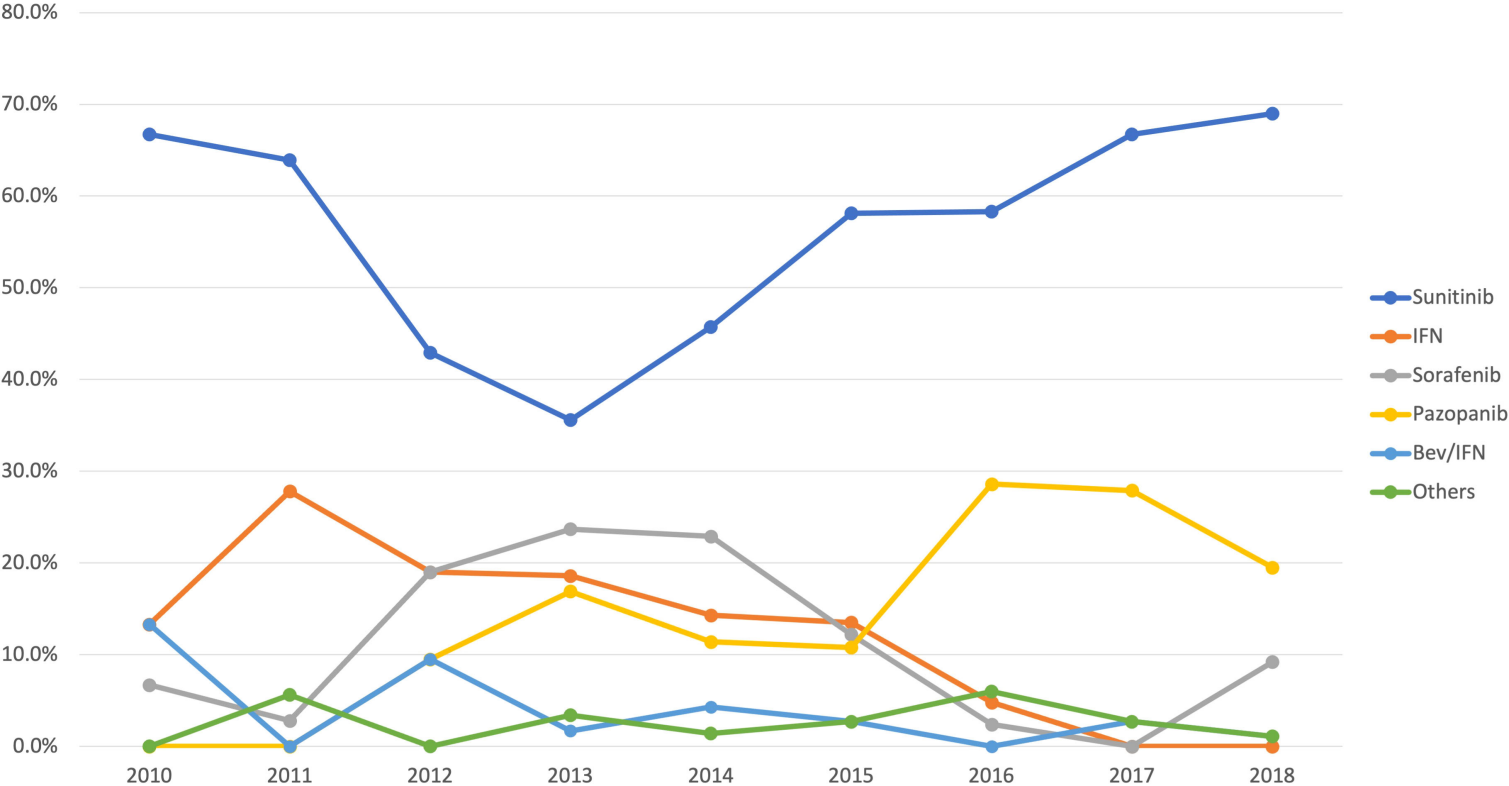
Distribution of first-line systemic therapy used by year.

**Figure 2:**
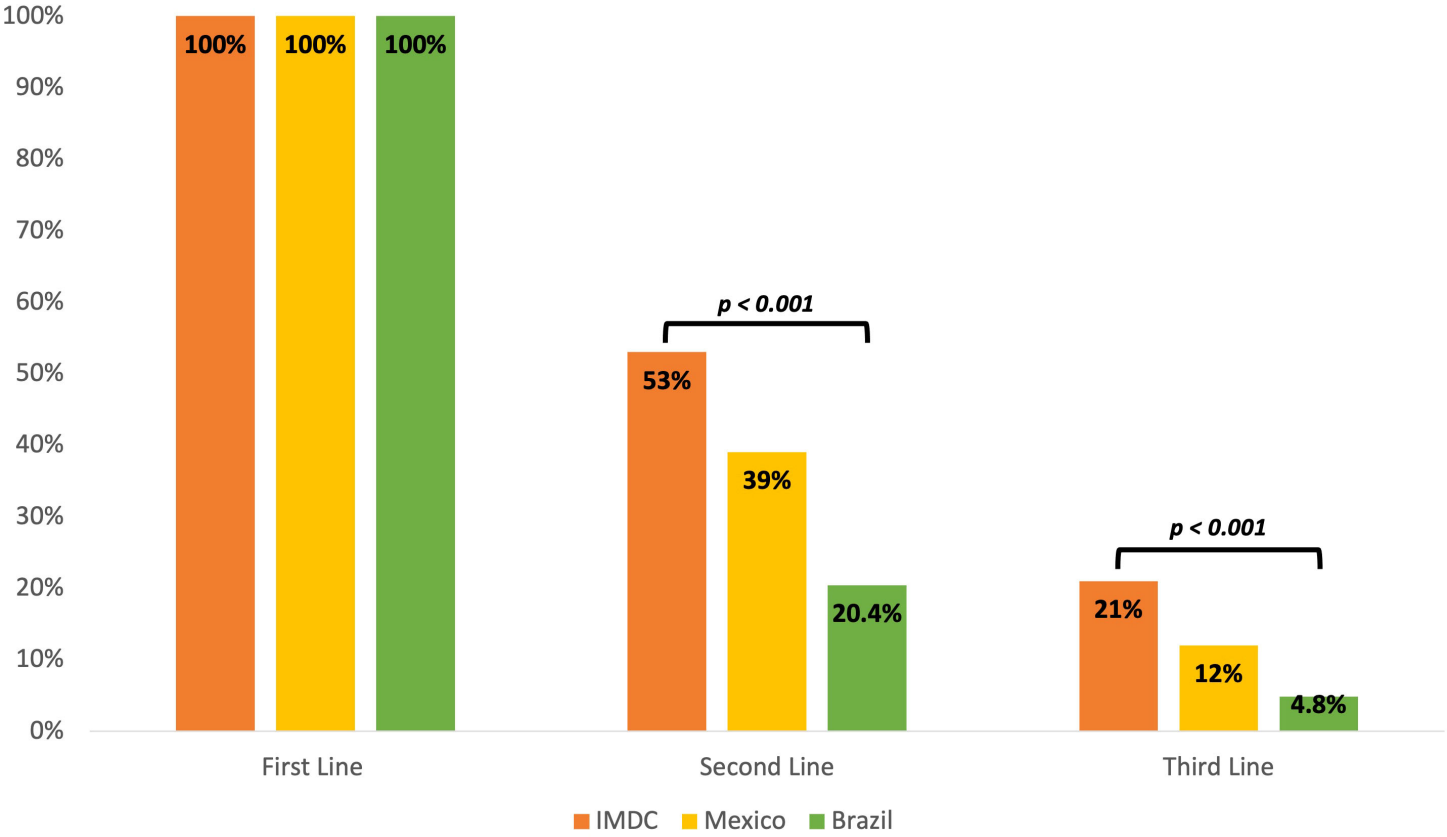
First-, second- and third- line regimen for mRCC comparing IMDC, Mexican, and Brazilian cohorts, among patients who started systemic treatment.

Details on therapeutic regimens according to year of therapy initiation for first-line treatment can be found in **Figure 1**. Comparison of second- and third-line therapies received in our cohort with those of the IMDC (20) and the largest Latin American cohort (Brazilian) (21) on mRCC is shown in **Figure 2**. Patients in our cohort were less likely to receive second- (53% vs 36%) and third-line (21% vs 10%) treatments as compared to the IMDC cohort (p<0.001).

### Overall survival

**Figure 3:**
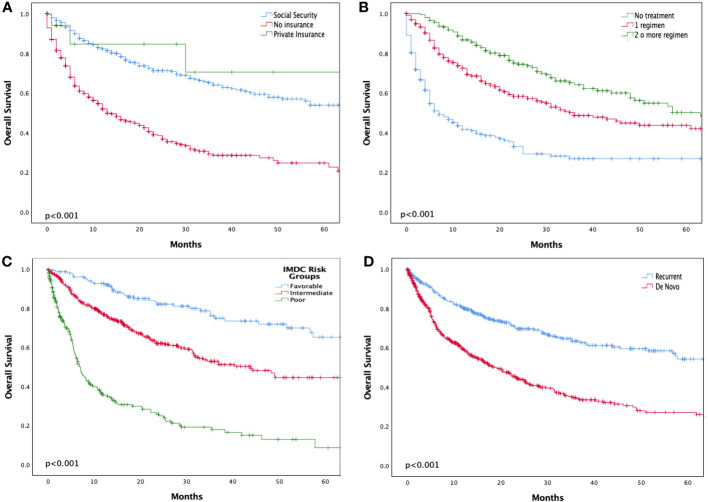
Kaplan-Meier survival curve of overall survival according to insurance status **(A)**, number of treatment regimens **(B)**, IMDC prognostic index **(C)**, and De novo metastatic disease **(D)**.

Median follow-up for the entire cohort was 86 months (range 57 – 114 months). Median OS for the overall population was 31.3 months (95% CI, 24.9 - 37.8). Median OS according to healthcare insurance was 13.9 months (95% CI, 10.6 - 17.2) for NI, 98.9 months (95% CI, 46.8 - 151.2) for SS, and 147.6 months (95% CI, 0 - 317.1) for PI (p < 0.001) (**Figure 3A**). In terms of systemic therapy access, median OS for patients who did not receive systemic treatment was 7.1 months (95% CI, 4.1 - 10.1), 35.1 months (95% CI, 25 - 45.1) for patients treated with one regimen, and 57.7 months (95% CI, 41.7 - 73.7) for those who received ≥2 lines of treatment (p < 0.001) (**Figure 3B**).

According to IMDC risk groups, median OS was not reached for the favorable risk subgroup, 43.8 months (95% CI, 32.3 - 55.4) for the intermediate risk subgroup, and 6.9 months (95% CI, 5.7 - 8.1) for the poor risk subgroup (p<0.001) (**Figure 3C**). According to disease presentation, median OS was significantly shorter in patients with *de novo* metastatic disease [18.6 months (95% CI, 14.6 - 22.6)] compared with recurrent metastatic disease (147.6 months; 95% CI, 28.5 - 266.6, p<0.001) (**Figure 3D**].

In the multivariate survival analysis clinical, treatment, and insurance factors were evaluated (**Table 3**). Clinical factors associated with poorer survival were the presence of brain metastases (HR 2.2 95%CI 1.58-3.1; p < 0.001) and sarcomatoid features (HR 1.7; 95% CI 1.19 - 2.42; p = 0.004). Favorable IMDC risk group was the only protective factor for OS (HR 0.51; 95% CI 0.34 - 0.77; p = 0.001). Among treatment-related factors the absence of systemic treatment was associated with inferior survival versus ≥ 1 lines of treatment (HR 2.02 95%CI 1.43-2.85; p < 0.001). Lastly, NI status was an independent factor for worse survival compared with SS or PI (HR 2.1; 95% CI, 1.52 - 2.88; p < 0.001).

**Table 3:** Multivariate analysis (Cox Regression) for OS.

Characteristics	Univariate cHR (95% CI)	p	Multivariate aHR (95% CI)	p
Biologic Factors				
Sex (Female vs Male)	0.97 (0.79-1.19)	0.782		
Age (≤ 65y vs >65)	1.05 (0.84-1.31)	0.675		
Sarcomatoid features	2.01(1.46-2.75)	**<0.001**	**1.7 (1.19-2.42)**	**0.004**
Sites of metastases (1 vs >1)	0.72 (0.58-0.88)	**0.001**	0.88 (0.67-1.17)	0.395
*De novo* vs recurrent disease	2.31 (1.86-2.88)	**<0.001**	0.96 (0.71-1.23)	0.769
Favorable IMDC risk	0.30 (0.21-0.42)	**<0.001**	**0.51 (0.34-0.77)**	**0.001**
Brain Metastases	1.53 (1.17-1.99)	**0.002**	**2.2 (1.58-3.1)**	**<0.001**
Bone Metastases	1.50 (1.22-1.84)	**<0.001**	**1.46 (1.13-1.89)**	**0.004**
Socioeconomic Factors				
No insurance	2.95 (2.39-3.65)	**<0.001**	**2.1 (1.52-2.88)**	**<0.001**
Foreigners	1.31 (1.05-1.63)	**0.018**	0.95 (0.7-1.28)	0.735
Therapeutic Factors				
Nephrectomy	0.36 (0.29-0.44)	**<0.001**	**0.58 (0.42-0.79)**	**0.001**
Metastasectomy	0.23 (0.14-0.36)	**<0.001**	**0.2 (0.11-0.36)**	**<0.001**
0 vs ≥ 1 lines of treatment	2.97 (2.43-3.64)	**<0.001**	**2.02 (1.43-2.85)**	**<0.001**

Bold values are those that reached statistical significance.

## Discussion

To the best of our knowledge, this is the first multicenter cohort of patients with mRCC in Mexico, and the second largest in Latin America (21). Several similarities in frequency of clinical characteristics were found between the IMDC external validation, Brazilian, and our cohorts, including clear cell histology, sarcomatoid features, and distribution of IMDC risk groups (20, 21). Although, the IMDC Prognostic Index has not yet been validated in the Mexican population, it appears that IMDC risk groups correlated with survival outcomes in our study and, therefore, endorses its role as a prognostic tool in our population. Remarkably, OS seemed to be longer for the patients in the good and intermediate risk subgroups in our study as compared to the patients in the IMDC cohort (20). This difference in survival outcomes might be possibly explained by the relatively high proportion of patients in our cohort with recurrent metastatic disease (87%), who might display a less aggressive tumor biology as compared to patients with *de novo* metastatic disease. Furthermore, previously described prognostic factors, including the presence of sarcomatoid pattern (22), brain metastases (23), bone metastases (24), number of systemic therapies (25), number of metastatic sites (26), and IMDC group risk (20, 22), were also statistically associated with worse survival in our cohort.

The type of treatments received across our study mirrors those of high- and upper-middle-income countries a decade ago, with sunitinib being the most common treatment prescribed, and pazopanib being more frequently prescribed since its approval in 2010 (21, 27). Strikingly, a higher proportion of patients in our cohort (36%) did not receive any systemic treatment when compared to the largest Latin American cohort (21%) (21). A probable reason for this finding may be that most of the patients included in our study were treated under the NI system, thus, financial constraints might have impeded them from accessing effective antineoplastic therapies. However, among patients who started a first-line of treatment, a higher percentage of patients in our cohort were able to receive second- and third-line therapies than in the Brazilian cohort (21). Nonetheless, all these proportions are inferior to those reported in the IMDC validation cohort (28), highlighting the major lack of access to effective cancer treatments in Latin America.

The most important finding of our study is the clear relationship between healthcare system inequities and survival. Unsurprisingly, healthcare insurance status was an independent prognostic factor for survival in the multivariate analysis. The impact of healthcare insurance on OS has also been described in other upper-middle- and high-income countries (29–32), and more recently, in other Latin American countries such as Brazil (21, 33). These data highlight the importance of access to treatment in an era during which the most effective cancer therapies are extremely expensive and cannot be systematically covered by healthcare systems in LMICs.

Limited access to effective, yet high-cost therapies for all cancer types, including RCC, is a common problem among LMICs, such as Mexico (34–36). In addition to the high prescription costs, which patients often cover through out-of-pocket expenses, access to standard of care cancer therapy may be further restricted by long delays in medications’ approval by local authorities in limited-resource settings, contrary to “fast-track” approvals issued by the U.S. Food and Drug Administration (FDA) and/or European Medicines Agency (EMA) (34, 36). Thus, without government reimbursements, insurance, or any specialized access programs in LMICs, a substantial number of patients are not able to access standard of care treatment and mortality continues to rise in limited-resource settings (35). To improve access to standard of care treatment of mRCC, pricing policies could be implemented to reduce the costs of cancer medicines within countries and across regions, as have been effected for other communicable diseases such as HIV (34, 37, 38).

Additionally, the treatment landscape of mRCC has changed dramatically in the last years (11, 14, 39) and in the present study, these new treatment paradigms and their impact on OS were underrepresented. Our data reflect the disparities present in many middle-income countries, where only a minority of the population with private healthcare insurance might have access to novel, effective treatments, whereas the majority of non-insured and/or SS patients are treated with non-standard therapeutics.

A feasible strategy to overcome barriers limiting access to effective cancer therapies may be the promotion and inclusion of patients from LMICs into clinical trials. Although clinical cancer research conducted in limited-resource settings is disproportionately low as compared to HICs (40, 41), the number of registered clinical trials has increased in all geographic regions, including Latin American and the Caribbean (42). Notably, Brazil was the top-performing country in Latin America, contributing to 41.8% of cancer-related articles published from 2010 to 2018, followed by Mexico (16.6%) and Argentina (12.9%) (43, 44). Although this option may allow patients to access novel agents, medical oncologists in Latin America often face multiple obstacles when trying to conduct clinical research, mainly regulatory and financial constraints (45). Funding for cancer research has improved in Brazil by the creation a successful program that allows tax deduction through donations to specific research projects and through patient advocacy groups raising funds for cancer research (43).

In Mexico, access to effective treatment strategies is further complicated by differences in drug availability and approved indications even within the public healthcare system, consequently, the choice of therapy and the number of available therapies may vary from one center to another. A subsequent analysis of treatment patterns in this cohort would be pertinent to contrast the newly established treatment options with the reality of a resource-constrained setting. Moreover, the centralized healthcare system in Mexico is also a factor that might have potentially impacted on the treatments received in our cohort, particularly for the NI patients subgroup, who more frequently need to travel long distances for medical care due to the lack of public healthcare cancer centers closer to their areas of residence. Unfortunately, if the emergence and approval of new, high-cost drugs continues increasing rapidly, the gap in access to standard of care treatments will continue to widen, especially in LMICs.

Some LMICs, including many countries in Latin America, have shifted their efforts towards creating universal healthcare systems to provide basic care for their previously uninsured populations (46). Although the healthcare system in Costa Rica has been acknowledged as an example of almost complete universal health care, the replication of this system to the rest of Latin America is difficult because, by contrast to its neighbors, Costa Rica has a history of political stability (47). Conversely, a major and current problem for the Mexican healthcare system is the lack of continuity across different government mandates. For example, in 2003 a major healthcare reform called “ Seguro Popular” was launched to address the low healthcare budget and unfair distribution of medical services in Mexico as well as to provide high-quality medical services for the uninsured population (48). Until December 2019, 40.6% of the Mexican population was covered by Seguro Popular (49), and management of the most common neoplasms, including cervical, breast, testicular, and prostate cancer was covered free of charge (50). Nonetheless, this reform was replaced in January 2020 by another healthcare reform (51), which was recently canceled in early 2023 (50). Thus, lack of continuity of effective healthcare programs in Mexico further contributes and augments healthcare disparities in our country.

The present study has several limitations, the foremost is its retrospective nature with its inherent biases and the heterogeneity in sample size between centers as well as PI, SS and NI subgroups. The number of PI patients was low, and comparisons should be interpreted with caution. However, the proportion of PI patients in our study mirrors that of the general Mexican population (52). Possibly, selection bias led the OS in patients with SS and PI in our cohort being higher than expected for a resource-constrained setting with limited access to the current standard of care therapies for mRCC, as many patients may have not been included because they were not candidates for active treatment or were referred only to palliative care services. Finally, the present study highlights the impact of treatment access on survival. National and international initiatives must be undertaken to ensure universal access to standard of care treatment in LMICs, minimizing the gap in survival opportunities between different populations.

## Conclusions

Our study represents the first multicenter cohort of patients with mRCC in Mexico, whose clinical and prognostic characteristics resembles those of large multinational studies. In addition to previously known adverse features associated with worse prognosis, the type of healthcare insurance was significantly associated with access to treatment and, subsequently, survival. Our results demonstrate that healthcare insurance status is a major prognostic factor for patients with mRCC in limited-resource settings such as Mexico, where access to effective treatment strategies is further complicated by differences in drugs’ availability and a fragmented healthcare system. Thus, the development and implementation of strategies aiming to reduce healthcare system disparities and to improve cancer care for all patients with mRCC in an era of highly effective and costly treatments are urgently needed.

## Data availability statement

The raw data supporting the conclusions of this article will be made available by the authors, without undue reservation.

## Ethics statement

The study was conducted in accordance with the Declaration of Helsinki and was approved by Institutional and Ethics Review Boards within all participating institutions: Instituto Nacional de Ciencias Médicas y Nutrición Salvador Zubirán, Centro Médico Nacional Siglo XXI, Centro Médico Nacional 20 Noviembre, Médica Sur, Instituto de Seguridad Social del Estado de México y Municipios, and Instituto Nacional de Cancerología. All participating institutions are part of a national research initiative, the Collaborative Oncology Research Group of Mexico (GCIMO), Genitourinary Oncology section, created and supported by the Mexican Society of Oncology (SMeO). The studies were conducted in accordance with the local legislation and institutional requirements. Written informed consent for participation was not required for this study in accordance with the national legislation and the institutional requirements.

## Author contributions

MB: Conception and design, Analysis and interpretation, Writing the manuscript, Critical revision of the article, Data collection, Provision of material, patients or resources, Statistical expertise, Literature search, Administrative, technical, or logistic support. YR-B: Conception and design, Analysis and interpretation, Writing the manuscript, Critical revision of the article, Data collection, Provision of material, patients or resources, Statistical expertise, Literature search, Administrative, technical or logistic support. AA-M: Analysis and interpretation, Writing the manuscript, Critical revision of the article, Data collection, Statistical expertise, Literature search. BS-O: Writing the manuscript, Critical revision of the article, Data collection, Literature search. AP-S: Writing the manuscript, Critical revision of the article, Data collection, Literature search. NM-I: Writing the manuscript, Critical revision of the article, Data collection, Literature search. FC-A: Conception and design, Analysis and interpretation, Writing the manuscript, Critical revision of the article, Data collection, Provision of material, patients or resources, Statistical expertise, Literature search, Administrative, technical or logistic support. AM-A: Conception and design, Analysis and interpretation, Critical revision of the article, Data collection, Provision of material, patients or resources, Literature search, Administrative, technical or logistic support. SR-R: Conception and design, Analysis and interpretation, Critical revision of the article, Data collection, Provision of material, patients or resources, Literature search, Administrative, technical or logistic support. FM-R: Writing the manuscript, Critical revision of the article, Data collection, Literature search. PP-P: Conception and design, Analysis and interpretation, Critical revision of the article, Data collection, Provision of material, patients or resources, Literature search, Administrative, technical or logistic support. MD-A: Conception and design, Analysis and interpretation, Critical revision of the article, Data collection, Provision of material, patients or resources, Literature search, Administrative, technical or logistic support. JR-M: Conception and design, Analysis and interpretation, Critical revision of the article, Data collection, Provision of material, patients or resources, Literature search, Administrative, technical or logistic support. SC-G: Conception and design, Analysis and interpretation, Critical revision of the article, Data collection, Provision of material, patients or resources, Literature search, Administrative, technical, or logistic support. BM-C: Writing the manuscript, Critical revision of the article, Data collection, Literature search. EL: Analysis and interpretation, Writing the manuscript, Critical revision of the article, Statistical expertise. NS-M: Conception and design, Analysis and interpretation, Critical revision of the article, Data collection, Provision of material, patients or resources, Literature search, Administrative, technical or logistic support. All authors contributed to the article and approved the submitted version.
